# Simultaneous Multi‐Slice Acceleration for Free‐Breathing Motion Corrected Late Gadolinium Enhancement Imaging

**DOI:** 10.1002/mrm.70200

**Published:** 2026-02-15

**Authors:** Grzegorz Tomasz Kowalik, Karl P. Kunze, Filippo Bosio, Peter Speier, Daniel Staeb, Radhouene Neji, Blanca Domenech‐Ximenos, Andrew Tyler, Reza Razavi, Muhummad Sohaib Nazir, Amedeo Chiribiri, Sébastien Roujol

**Affiliations:** ^1^ Faculty of Life Sciences and Medicine, School of Biomedical Engineering and Imaging Sciences King's College London London UK; ^2^ MR Research Collaborations, Siemens Healthcare Limited Camberley UK; ^3^ Cardiovascular Predevelopment, Siemens Healthcare GmbH Erlangen Germany; ^4^ MR Research Collaborations, Siemens Healthcare Limited Melbourne Australia; ^5^ Department of Radiology Hospital Clínic de Barcelona Barcelona Spain; ^6^ Royal Brompton Hospital, Part of Guy's and St Thomas Hospital NHS Foundation Trust London UK

**Keywords:** free‐breathing, late gadolinium enhancement, motion correction, simultaneous multi‐slice

## Abstract

**Purpose:**

The objective of this study was to develop and evaluate an accelerated free‐breathing motion corrected and averaged late gadolinium enhancement MRI (FB PSIR‐MoCo LGE) protocols.

**Methods:**

Simultaneous multi‐slice (SMS) bSSFP imaging with GC‐LOLA correction and a multiband factor of two was implemented into a FB PSIR‐MoCo LGE sequence with phase sensitive inversion recovery (PSIR) reconstruction. Two FB PSIR‐MoCo LGE protocols were acquired in 26 patients referred for clinical cardiac MRI: (1) SMS‐accelerated, and (2) non‐SMS reference. Myocardial sharpness, scar‐to‐blood CNR estimate (CNRe), scar‐to‐healthy myocardium CNRe, blood‐to‐healthy myocardium CNRe, and scar volume were assessed. Scar assessment was conducted in all LGE‐positive patients (*N* = 10). Image quality (4‐point scale: 1—*poor/non diagnostic*, 4—*excellent/diagnostic*) was assessed by consensus of two readers.

**Results:**

SMS resulted in a twofold reduction of acquisition time (reference: 3.7 ± 0.9 min, proposed: 1.9 ± 0.6 min, *p* < 0.001). Although both approaches resulted in good to excellent image quality, the proposed approach resulted in slightly reduced image quality scores (reference: 3.64 ± 0.36 vs. proposed: 3.46 ± 0.36, *p* = 0.001) and modest (∼20%) reduction in CNRe. Myocardial sharpness measures showed no statistically significant differences between the two approaches (0.37 ± 0.06 vs. 0.37 ± 0.05 mm^−1^, *p* = 0.84). Importantly, both approaches were in excellent agreement for scar volume assessment (bias = 0.48 ± 0.86 mL, *R* ^2^ = 0.996).

**Conclusion:**

The proposed SMS FB PSIR‐MoCo LGE protocol reduced scan time by a factor of 2 when 16 slices were obtained, with minimal impact on image quality and CNRe, and no differences in myocardial sharpness. Importantly, it provided excellent agreement for myocardial scar assessment, in comparison to the reference FB PSIR‐MoCo LGE.

## 1 Introduction

Late gadolinium enhancement (LGE) MRI [1, 2] is the clinical gold standard technique for noninvasive imaging of myocardial scar [3]. The location, size, transmural extent, and pattern of LGE has high diagnostic and prognostic value in ischemic [4] and nonischemic heart diseases [5]. LGE MRI is commonly acquired 10–20 min after injection of a gadolinium‐based contrast agent using an inversion recovery approach [3]. The inversion time (TI) is selected from a prior “TI scout” to null the signal from healthy myocardium. Phase‐sensitive inversion recovery (PSIR) reconstruction [6] enables to preserve the signal polarity of tissues, and is often applied to LGE‐MRI to improve the contrast reliability in the presence of imperfect TI selection. LGE MRI commonly employs a breath‐hold 2D segmented imaging technique, where one slice is acquired per breath‐hold. Full ventricular coverage thus requires repeated breath‐hold acquisitions, which remains challenging in patients with impaired cognitive function or limited breath‐hold capabilities.

Several imaging techniques have been proposed as alternatives for patients with limited breath‐hold capabilities. Single breath‐hold LGE protocol have been developed to minimize breath‐hold requirements [7, 8, 9], but may still be unfeasible in some patients. Alternatively, free‐breathing 3D LGE sequences with respiratory navigation have been developed [10], but can be limited by long scan time especially when acquired at high spatial resolution [11, 12], and/or limited image quality due to increased sensitivity to respiratory motion artifacts, varying heartrate and changes to contrast concentration in myocardial scar during the scan [10, 11].

Free‐Breathing motion corrected and averaged 2D LGE (FB PSIR‐MoCo LGE) MRI is a robust clinical alternative in patients unable to breath‐hold [13, 14, 15]. It is based on repeated single shot acquisitions of the same slice, which are then co‐registered and averaged for enhanced quality. Eight single shot images are commonly acquired per slice, which can prolong the overall cardiac MRI examination. Therefore, reduction of the LGE acquisition duration, and therefore overall examination time, without compromising on image quality would be desirable to minimize cost, facilitate wider adoption and allow higher patient throughput [16].

Simultaneous multi‐slice (SMS) is a promising acceleration technique to reduce examination time without degrading spatial resolution. It uses multiband pulses to simultaneously excite multiple 2D slices. The simultaneously excited slices can be shifted with respect to each other in image space, using Controlled Aliasing in Parallel Imaging Results in Higher Acceleration (CAIPIRINHA) encoding [17], to facilitate image reconstruction using parallel imaging techniques. This is achieved by applying slice‐dependent RF phase cycling. This approach can be applied with bSSFP using an adjusted phase cycling scheme [18] and gradient‐controlled local Larmor adjustment (GC‐LOLA) [19] to correct for misaligned frequency responses across slices. Although this approach has been successfully demonstrated to improve spatial coverage of myocardial perfusion [20, 21, 22, 23, 24], its potential to accelerate LGE remains to be demonstrated.

In this study, we sought to evaluate the potential of SMS bSSFP imaging to reduce scan time of FB PSIR‐MoCo LGE protocol. This technique is compared to a reference single band FB PSIR‐MoCo LGE sequence in patients.

## 2 Methods

### 2.1 SMS FB PSIR‐MoCo LGE

The FB PSIR‐MoCo LGE research sequence consisted of a repeated (*N* = 8) inversion‐recovery ECG‐triggered single shot acquisition of the same slices under free‐breathing conditions. A two RR inversion recovery scheme was employed to enable PSIR reconstruction. Cartesian k‐space sampling was used. SMS‐bSSFP and GC‐LOLA using a multiband factor of two were implemented into this FB PSIR‐MoCo LGE protocol. The previously developed SMS bSSFP solution [19] was used in this study where slice‐specific RF phase cycling (phase increment of *π*/2 for slice 1 and −π/2 for slice 2) for CAIPIRINHA encoding resulting in a half‐FOV shift between the two slices in the phase‐encode direction [17, 18]. The resulting off‐centered frequency response profiles in both slices were corrected using GC‐LOLA based on an additional gradient in the slice direction [19]. In this implementation, the acceleration problem was transformed from a two‐dimensional problem (twofold slices and *N*‐fold in‐plane) into a one‐dimensional problem (2 × *N*‐fold in‐plane), as outlined in earlier work [25]. Here, N represents the desired in‐plane acceleration factor. This was accomplished by doubling the phase field‐of‐view (FOV), applying the above RF phase cycling to distribute the two slices across the expanded FOV, and implementing an in‐plane acceleration of 2 × *N* using the system's in‐built T‐GRAPPA undersampling and reconstruction framework [25], as previously described [19]. As a result, the shifted slice is reconstructed within the oversampled FOV and the two slices are then separated using a straightforward FOV partitioning step.

The multiple (*N* = 8) images acquired for each slice were co‐registered and averaged to suppress any residual artifacts and improve overall image quality. The following motion correction steps were applied individually for each slice. First, the two images with the most dissimilar motion states from the rest of the series were discarded. To this end, the center‐of‐mass point was calculated for each image of the time series. An initial average center‐of‐mass was calculated based on the eight images. This was further updated by taking the average of the four closest points to it. The six images with their center‐of‐mass closest to the final average were co‐registered using a non‐rigid registration approach [26, 27], and averaged to generate the final reconstruction image.

### 2.2 Experimental Evaluation

All imaging was performed on a 1.5 T scanner (MAGNETOM Aera, Siemens Healthineers AG, Erlangen, Germany) using an 18‐element body coil and a 32‐channel spine coil. Twenty five patients (15 male/10 female, age: 58 ± 17 years old) referred for a clinical cardiac MRI examination with contrast were prospectively recruited. The study was approved by the National Research Ethics Service (15/NS/0030) with written informed consent obtained from all patients for inclusion in the study and additional imaging during their clinical MRI examination.

### 2.3 Imaging Protocol

Each subject was scanned using the proposed SMS FB PSIR‐MoCo LGE sequence and a reference single‐band FB PSIR‐MoCo LGE sequence. Both sequences were acquired in a randomized order across subjects, ∼20 min after intravenous injection of 0.2 mmol/kg gadobutrol (Gadovist, Bayer Pharmaceuticals, Berlin, Germany). Both sequences used an inversion‐recovery ECG‐triggered single shot acquisition with bSSFP acquired in the short axis orientation, with the following imaging parameters: TR/TE: 2.71/1.15 ms, flip angle: 45°, FOV: 380 × 380 mm^2^, acquired resolution: 1.48 × 1.98 mm^2^, slice thickness: 8 mm, bandwidth: 1085 Hz/px, and acquisition window: 208 ms. SMS used an overall in‐plane acceleration factor of 5 and a phase oversampling factor of 2, which corresponds to a nominal in‐plane acceleration factor of 2.5. The reference sequence used an in‐plane acceleration factor of 3, a phase oversampling factor of 1.2, and image cropping to the prescribed FOV, which is also roughly equivalent to a nominal in‐plane acceleration factor of 2.5, although with a slightly increase g‐factor penalty. Both sequences thus had an equivalent in‐plane acceleration factor of 2.5 with a matched number of acquired k‐space lines and matched effective aliasing‐free FOV. A multiband factor of two was used for the SMS protocol. Therefore, the total acceleration factor was 5 for SMS and 2.5 for standard. To accommodate larger patients the FOV was adjusted up to a maximum of 460 mm depending on patient's anatomy. A short axis stack of 16 slices was prescribed to achieve full LV coverage without slice gaps in all patients. For the SMS acquisition, simultaneously excited slices had a slice gap equivalent to 8 slice thickness (e.g., SMS slices were 1–9, 2–10, …, 8–16). Eight repeated measurements of each slice were collected. This resulted in total scan time equivalent to 128 heartbeats for the proposed SMS sequence and 256 for the reference sequence, accounting for the two RR acquisition scheme used for PSIR reconstruction.

### 2.4 Data Analysis

To confirm the proposed approach does not introduce any additional blurring, myocardial sharpness was first computed for both sequences in all patients. Healthy myocardium to LV blood pool CNR estimates (CNRe) was also evaluated in all patients. Furthermore, healthy myocardium to scar CNRe, LV blood pool to scar CNRe and scar volumes were measured for all LGE positive patients (*N* = 10). These quantitative metrics were computed on PSIR images using MATLAB R2023b (The MathWorks Inc., Natick, MA).

The myocardial sharpness index was calculated as previously described [28, 29] for each slice. Briefly, two curves with closely spaced points were drawn on each side of the septal blood‐myocardium interface. A signal intensity profile was generated for each point on the myocardial curve and its closest point on the blood pool curve. The sharpness index of a signal intensity profile was defined as the reciprocal of the distance for the signal to drop from 80% to 20% of the signal range on that signal profile. The myocardial sharpness index of a slice was measured as the average sharpness index measured for all signal profiles. Finally, an average myocardial sharpness across all slices was measured for each subject.

Healthy myocardium to LV blood pool CNRe was assessed in all LV slices. Healthy myocardium to scar CNRe and LV blood pool to scar CNRe were evaluated in LGE positive slices. The signal from healthy myocardium, LV blood pool, and scar was measured as the mean signal of a manually delineated region of interest (ROI) over the relevant anatomy/tissue type on a slice‐basis. The noise was also estimated for each slice as follows. The noise estimate $\left(N_{e}\right)$ was approximated from the difference image of the two last motion‐corrected LGE images (i.e., before averaging) as $N_{e}=\frac{\sigma_{d}}{\sqrt{2}\sqrt{6}}$ where $\sigma_{d}$ is the signal standard deviation from the blood pool ROI. The scaling factors ($\sqrt{2}$ and $\sqrt{6}$) account for the doubled noise variance in the difference image and the averaging process (of six single‐shot LGE images) used to create the final LGE image, respectively.

Scar volume was measured from manual delineation of the scar area in each slice.

Subjective assessment of LGE magnitude image quality was also assessed in consensus by two readers (A.C. and M.S.N. with 17 and 12 years of CMR experience, respectively). DICOM images were visualized using Weasis software (N. Roduit, Weasis DICOM viewer, Version 4.2.0.). The CMR readers were blinded from patient information. Images obtained with each method were presented to the readers in a random order. All textual information that could reveal the acquisition method was hidden for this analysis. However, due to inherent small differences in SNR between methods, the analysis was not fully blinded to the acquisition type. Image quality was assessed using a 4‐point Likert scale: 1—*poor/non diagnostic*, 2—*major artifacts*/*diagnostic*, 3—*minor artifacts*/*diagnostic*, 4—*excellent*/*diagnostic*. Scores were collected for each slice of the short axis stack containing the left ventricle. The percentage of slices with each quality score was calculated. An average image quality score was also derived for each subject. The presence of scar on PSIR images was assessed for each slice and each of the 16 standardized AHA myocardial segments.

### 2.5 Statistical Analysis

Results are presented as mean ± standard deviation. Myocardial sharpness index, CNRe, and acquisition time were compared between both techniques using paired two‐tailed *t*‐test. The Wilcoxon signed rank test was used to compare image quality scores and scar volume obtained with both methods. A *p* < 0.05 was considered statistically significant.

## 3 Results

Both sequences were successfully acquired in all patients. Example PSIR image series, corresponding motion corrected images, and final average images are shown for the proposed SMS FB PSIR‐MoCo LGE sequence in Figure S1. The applied co‐registration algorithm successfully morphed the image series enabling residual artifact suppression and improved image quality through signal averaging.

**FIGURE 1:**
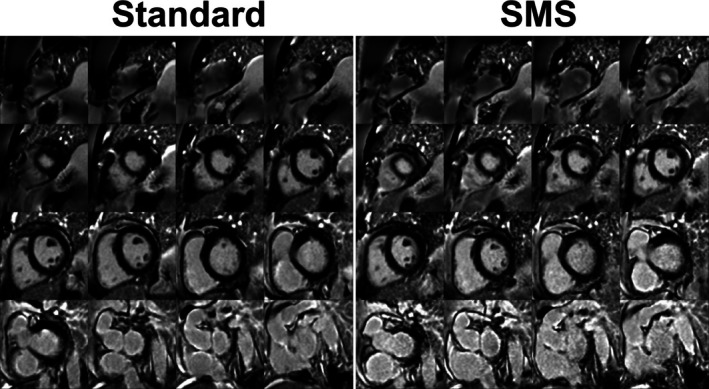
Examples of PSIR images obtained with the reference and SMS sequences in one patient for all slices.

**FIGURE 2:**
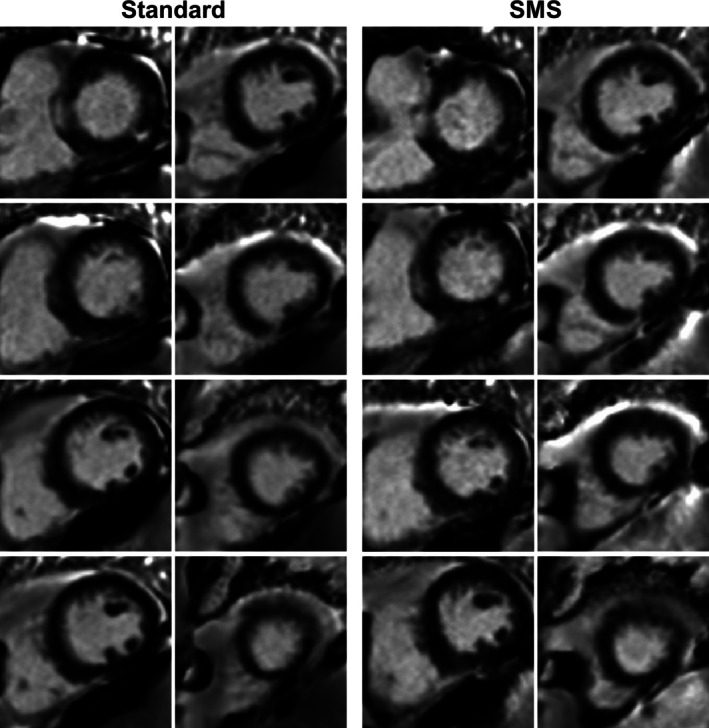
PSIR LGE images obtained with both reference and SMS sequences for a patient with positive LGE in basal and mid inferolateral segments.

**FIGURE 3:**
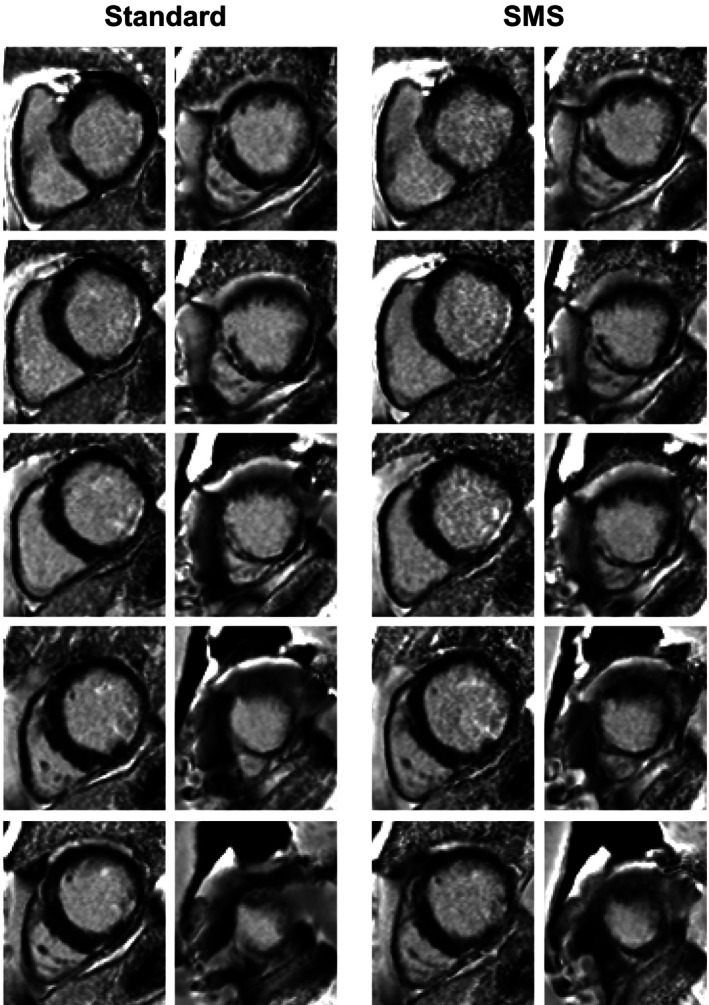
PSIR LGE images obtained with both reference and SMS sequences for a patient with positive LGE in basal inferolateral and mid/apical segments.

Examples of PSIR imaging results for all slices acquired with both sequences in one patient are shown in Figure 1. Excellent visually comparable image quality and image sharpness can be observed in both techniques across all slices. Two LGE positive patients are shown in Figures 2 and 3. Myocardial scarring can be observed in the same myocardial segments using both techniques for each patient: basal and mid inferolateral myocardial segments (Figure 2) and basal inferolateral and mid/apical septal segments (Figure 3). The scar territory and pattern are well defined in both series and matches well. Image quality was also excellent in these patients across all slices.

**FIGURE 4:**
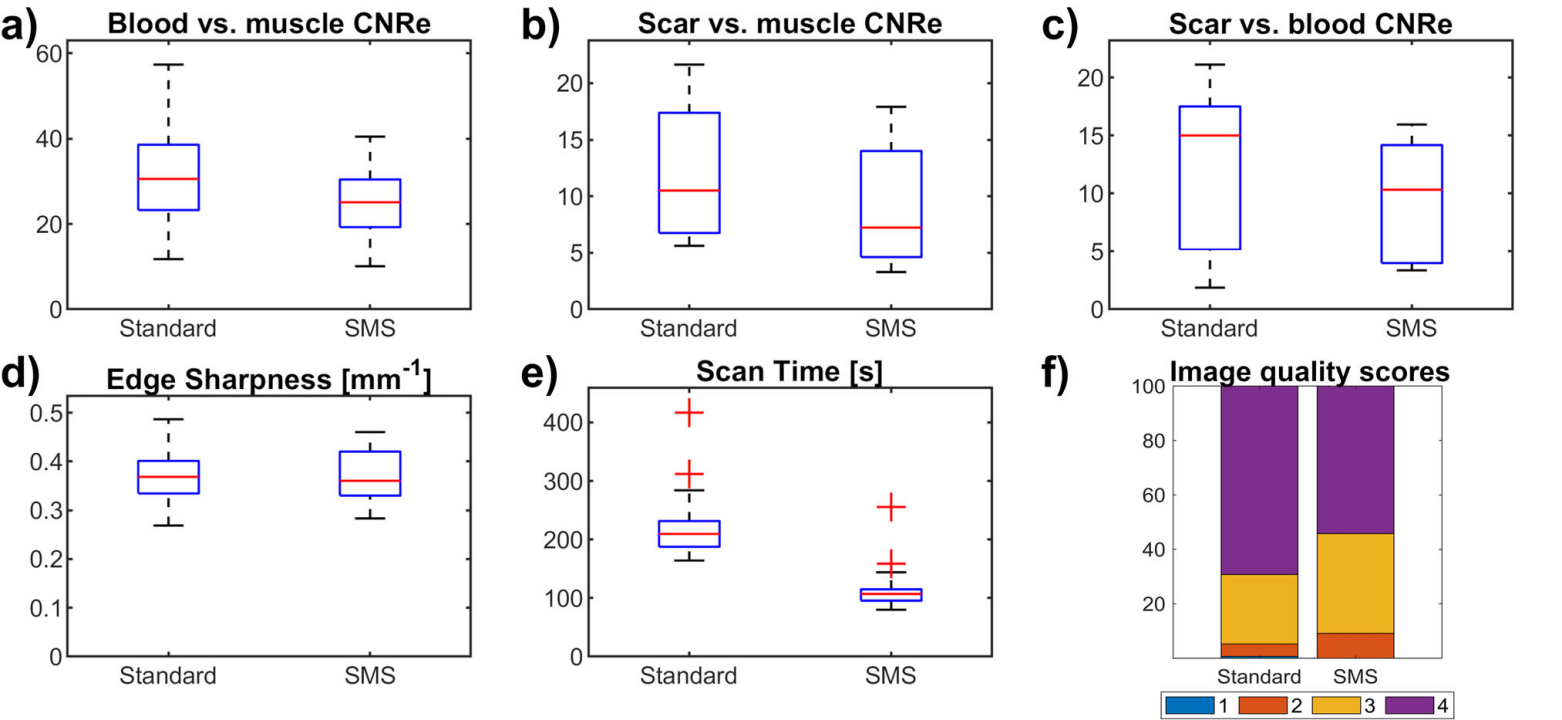
Quantitative and qualitative assessment of image quality metrics and scan time. LV blood pool‐to‐healthy myocardium CNRe (a), scar‐to‐healthy myocardium CNRe (b), scar‐to‐LV blood pool CNRe (c), edge sharpness (d), scan time (e), and image quality scores (f) are shown for both the reference and the proposed sequence. Likert scale used in the image quality assessment: 1—*poor*/*non diagnostic*, 2—*major artifacts*/*diagnostic*, 3—*minor artifacts*/*diagnostic*, and 4—*excellent*/*diagnostic*.

Quantitative metrics of the patient analysis are shown in Figure 4a–e. Across all patients, the proposed SMS protocol resulted in a twofold reduction of acquisition time (1.9 ± 0.6 vs. 3.7 ± 0.9 min [reference], *p* < 0.001). A small noise estimate amplification of ∼20% was obtained using the SMS approach as denoted by a slightly reduced healthy myocardium to blood CNRe (25 ± 8 vs. 31 ± 10 [reference], *p* < 0.001), healthy myocardium to scar CNRe (9 ± 6 vs. 12 ± 6 [reference], *p* < 0.03), and blood to scar CNRe (10 ± 5 vs. 12 ± 7 [reference], *p* = 0.08). There was no statistically significant difference in terms of myocardial sharpness (0.37 ± 0.05 vs. 0.37 ± 0.06 mm^−1^, *p* = 0.84) between the proposed and reference approaches, respectively.

Image quality scores are shown in Figure 4f. Both approaches provided good‐to‐excellent image quality scores. However, the reference approach led to slightly higher overall image quality scores than SMS (proposed: 3.46 ± 0.36 vs. standard: 3.64 ± 0.36, *p* = 0.001). The rate of diagnostic images (image quality score > 1) was 100% for SMS and > 99% for the reference.

**FIGURE 5:**
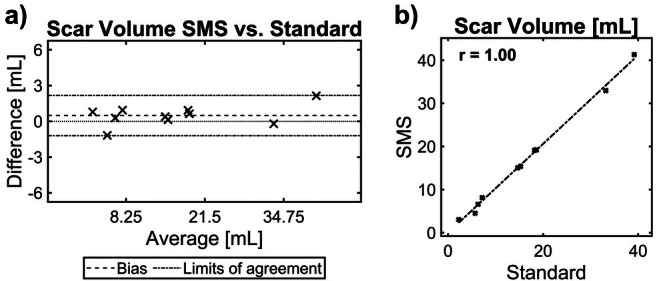
Myocardial scar volume assessment. Bland–Altman analysis (a) and linear regression plot (b) show excellent agreement between the reference and proposed approach for measuring myocardial scar volume.

Both sequences were not different for scar volume assessment (SMS: median 14.9 mL, IQR 12.3 mL; reference: median 15.2 mL, IQR 12.6 mL, *p* = 0.1). Linear regression analysis (*R* ^2^ = 0.996; with *p* < 0.001) (Figure 5a) and Bland–Altman analysis (bias = 0.48 ± 0.86 mL with a narrow width of 95% limits of agreement of 3.4 mL) (Figure 5b) showed excellent agreement between the two sequences for measuring myocardial scar volume.

## 4 Discussion

In this study, we demonstrated the clinical feasibility of SMS for accelerating standard FB PSIR‐MoCo LGE protocol. This SMS PSIR‐MoCo LGE protocol halved the acquisition time, for complete LV coverage, compared to the non‐SMS protocol. It resulted in a modest (∼20%) reduction in CNRe, minimal reduction of perceived image quality (∼5%), and no impact on myocardial sharpness. Importantly, the proposed approach was in excellent agreement with the reference approach for scar volume assessment.

A constant stack of 16 slices was used in this study to ensure full LV coverage, including in patients with larger hearts. For SMS imaging, a slice gap equal to eight slices (i.e., half the number of the prescribed slices) was introduced to maximize coil sensitivity differences of simultaneously excited slices. In practice, the number of slices could be reduced based on heart size, which would reduce the slice gap between simultaneously excited slices. Although the quality of SMS imaging may reduce as the slice gap is smaller, previous studies in CMR perfusion have shown that SMS can work well even with small slice gaps (< 1–2× slice thickness) [20, 21, 22, 23, 24]. Future studies will thus be necessary to fully characterize the impact on smaller slice gaps on the image quality of the proposed approach.

Expanding the proposed sequence to a higher multiband factor holds promise for further accelerating data acquisition and reducing scan time. Although the use of T‐GRAPPA image reconstruction with an overall acceleration factor of 5 was successful in this study, higher acceleration factors remain to be investigated, and are expected to suffer from an increased g‐factor penalty and greater reconstruction artifacts. Alternative acquisition schemes and or reconstruction strategies may become necessary in this context. SMS bSSFP with a multiband factor of 3 and high overall accelerator factor (up to 15) combined with a pseudo‐random undersampling pattern and compressed sensing reconstruction with temporal regularization was successfully demonstrated for CMR perfusion imaging [23, 24]. Compressed sensing with integrated motion correction was also proposed in several studies to improve robustness against respiratory motion [30]. However, the translation of such approach to the current application may suffer from the reduced number of available temporal frames. Alternatively, deep learning approaches have shown great potential for the reconstruction of highly accelerated SMS acquisition and may represent a promising solution to further accelerate the current sequence [31, 32, 33, 34].

RF‐CAIPI with GC‐LOLA correction was used for SMS acquisition. The blipped‐CAIPI [35] technique is an alternative approach for SMS bSSFP, where k‐space modulation is achieved using slice gradient blips. However, blipped‐CAIPI may be associated with higher fat signal leakage between simultaneously excited slices [30] and higher sensitivity to eddy current artifacts [19]. The comparison of both approaches will require further investigation.

FB PSIR‐MoCo LGE has been demonstrated in several large clinical studies to offer reduced scan time (3–4 min for a full short axis stack vs. 6–7 min for standard breath‐hold protocols), higher image quality, higher reader confidence and reproducibility, and no degradation of diagnostic performance [15, 36, 37, 38]. The scan time of the reference FB PSIR‐MoCo LGE used in this work is consistent with these prior studies. The proposed SMS acceleration provides a further twofold reduction of scan time leading to a complete LGE short axis stack acquired in less than 2 min. This approach thus has the potential to reduce CMR examination time which could lead to cost saving for healthcare systems and increased patient throughput [3]. Alternatively, the proposed acceleration approach could be used to acquire additional LGE contrasts (such as both bright blood and black blood LGE contrasts) or additional MR‐biomarkers without prolonging current CMR examination times. It could also enable LGE with higher spatial resolution and coverage. While standard LGE protocols often include a slice gap, the proposed approach may enable full LV coverage with potentially increased spatial resolution without scan time penalty.

This study has several limitations. First, the sample size of this study is small. However, the aim of the study was to demonstrate the clinical feasibility of SMS to accelerate a FB PSIR‐MoCo LGE protocol commonly used in clinical practice. Further studies in a larger patient cohort are now warranted. Second, only a limited number of patients were LGE positive, which has prevented to conduct a more comprehensive analysis of scar patterns such as scar transmurality level. A larger study will also be needed to compare scar patterns between the two protocols. Third, no direct comparison was made with a breath‐hold LGE protocol. However, the performance and benefit of the reference FB PSIR‐MoCo LGE protocol over a breath‐hold LGE protocol has previously been established [13]. Fourth, the reference FB PSIR‐MoCo LGE employed in this study used an effective in‐plane GRAPPA acceleration factor of 2.5 (only possible using a phase oversampling factor of 1.2, and a prescribed in‐plane GRAPPA factor of 3). This slightly differs from standard FB PSIR‐MoCo LGE sequences used clinically, where a GRAPPA acceleration factor of 2 is commonly used. This choice was motivated to ensure a matched effective in‐plane acceleration factor with SMS, since the overall SMS acceleration factor is constrained to an odd number in the current implementation (with thus an in‐plane acceleration factor limited to: 1.5, 2.5, 3.5, etc.). Fifth, although all quantitative metrics were assessed on PSIR images, subjective image quality was evaluated on magnitude images. However, image quality is not expected to differ between the magnitude and PSIR reconstructions. Lastly, the CMR readers were not fully blinded to the acquisition type as some of the image features (especially SNR) were slightly different between the two techniques.

## 5 Conclusion

The proposed SMS FB PSIR‐MoCo LGE protocol reduced scan time by a factor of two when 16 slices were obtained, with minimal impact on image quality and CNRe, and no differences in myocardial sharpness. Importantly, it provided excellent agreement for myocardial scar assessment, in comparison to the reference FB PSIR‐MoCo LGE.

## Funding

This study was supported by the Biomedical Research Centre at Guy's and St Thomas' National Health Service (NHS) Foundation Trust; British Heart Foundation (BHF), Grant/Award Numbers: PG/19/11/34243, PG/21/10539; Engineering and Physical Sciences Research Council (EPSRC), Grant/Award Number: EP/R010935/1; King's College London; Innovate UK, Grant/Award Number: 68539; and National Institute for Health Research (NIHR). This research was funded in whole, or in part, by the Wellcome Trust, Grant/Award Number: WT 203148/Z/16/Z. For the purpose of open access, the author has applied a CC BY public copyright license to any Author Accepted Manuscript version arising from this submission.

## Conflicts of Interest

Karl P. Kunze, Peter Speier, and Daniel Staeb were SIEMENS employees at the time of the work on this project. The other authors declare no conflicts of interest.

## Supporting information


**Figure S1:** LGE‐SMS PSIR‐MoCo image processing. The eight NSA are first reconstructed with T‐GRAPPA (pre‐MoCo). The six images with closest centre of mass are co‐registered for the following Motion Correction (MoCo). The corrected signal repetitions are averaged to create the final imaging result (averaged). The motion maps from magnitude images are re‐used in the PSIR image processing.
