# Supplementary material for: Simultaneous Multi‐Slice Acceleration for Free‐Breathing Motion Corrected Late Gadolinium Enhancement Imaging
Source: Magn Reson Med. 2026 Feb 15;95(6):3333–40. doi: 10.1002/mrm.70200 (PMC13049237; doi:10.1002/mrm.70200)
Supplement: Supplementary file 1 — Figure S1: LGE‐SMS PSIR‐MoCo image processing. The eight NSA are first reconstructed with T‐GRAPPA (pre‐MoCo). The six images with closest centre of mass are co‐registered for the following Motion Correction (MoCo). The corrected signal repetitions are averaged to create the final imaging result (averaged). The motion maps from magnitude images are re‐used in the PSIR image processing. [file MRM-95-3333-s001.docx]

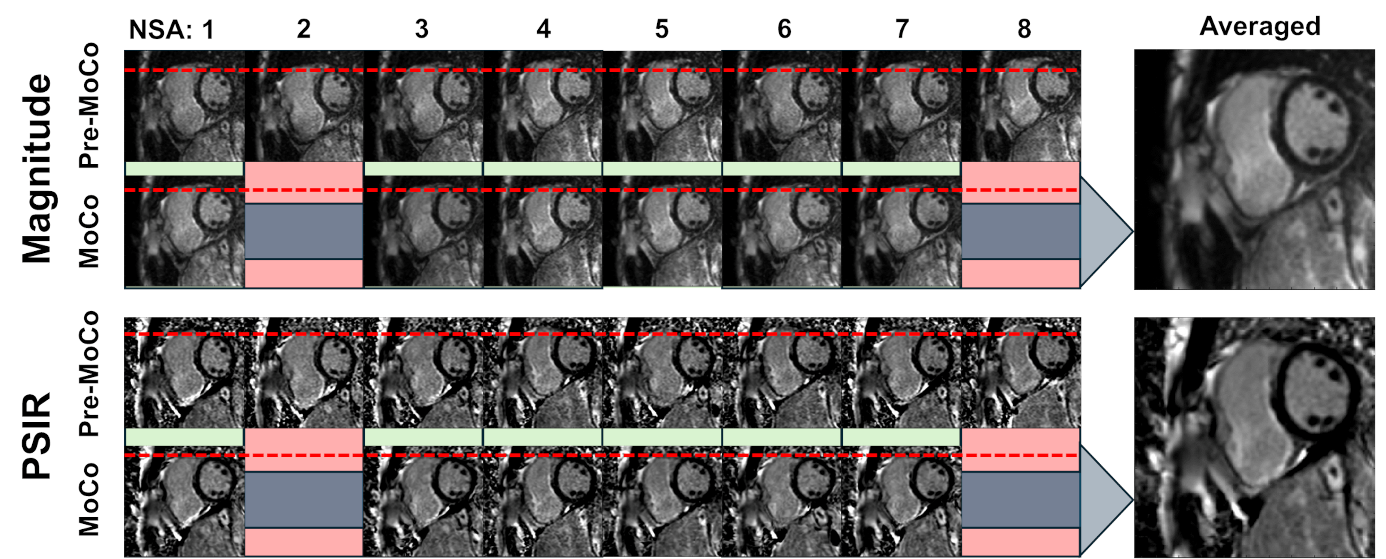
**Figure S1.** LGE-SMS PSIR-MoCo image processing. The 8 NSA are first reconstructed with T-GRAPPA (pre-MoCo). The 6 images with closest centre of mass are co-registered for the following Motion Correction (MoCo). The corrected signal repetitions are averaged to create the final imaging result (Averaged). The motion maps from magnitude images are re-used in the PSIR image processing.
